# Contribution of the Cognitive Approach to Language Assessment to the Differential Diagnosis of Primary Progressive Aphasia

**DOI:** 10.3390/brainsci11060815

**Published:** 2021-06-19

**Authors:** Joël Macoir, Annie Légaré, Monica Lavoie

**Affiliations:** 1 Département de Réadaptation, Faculté de Médecine, Université Laval, Québec, QC G1V 0A6, Canada; annie.legare@fmed.ulaval.ca; 2 Centre de Recherche CERVO (CERVO Brain Research Centre), Québec, QC G1J 2G3, Canada; 3 Chaire de Recherche sur les Aphasies Primaires Progressives—Fondation de la Famille Lemaire, Québec, QC G1J 1Z4, Canada; monica.lavoie.1@ulaval.ca

**Keywords:** primary progressive aphasia, assessment, diagnosis, cognitive approach, dementia

## Abstract

Diagnosis of primary progressive aphasia (PPA) is essentially based on the identification of progressive impairment of language abilities while other cognitive functions are preserved. The three variants of PPA are characterized by core and supportive clinical features related to the presence or absence of language impairment in different linguistic domains. In this article, we review the cognitive neuropsychological approach to the assessment of PPA and its contribution to the differential diagnosis of the three variants. The main advantage of this assessment approach is that it goes beyond the mere description and classification of clinical syndromes and identifies impaired and preserved cognitive and linguistic components and processes. The article is structured according to the main language domains: spoken production, language comprehension, and written language. Each section includes a brief description of the cognitive processes involved in the assessment tasks, followed by a discussion of typical characteristics for each PPA variant and common pitfalls in the interpretation of the results. In addition, the clinical benefit of the cognitive neuropsychological approach for the behavioral management of PPA is briefly sketched out in the conclusion.

## 1. Introduction

Dementia is a common condition that mainly occurs in older people. It is characterized by a decline in cognitive functioning that is severe enough to impact activities of daily living and social functioning [1]. The loss of cognitive functioning in dementia may affect long- and short-term memory, attention, visual perception, executive functions, motor planning and execution, problem solving, and language [2]. Dementia can be caused by a wide variety of pathological entities, including Alzheimer’s disease, which is the most common one. Other types of dementia include vascular dementia, dementia in atypical parkinsonian syndromes, such as Lewy body dementia and corticobasal degeneration, and frontotemporal dementia [3]. They are not only commonly associated with episodic memory impairment but also usually characterized by language deficits that may affect word and sentence comprehension and production abilities [4]. Clinical language profiles that are generally associated with common forms of dementia have been described, some in more detail than others. Neurolinguistic studies go beyond the mere description of symptoms to identify the functional localization of impaired and preserved linguistic processes in dementia.

Primary progressive aphasia (PPA) is a neurodegenerative syndrome associated with atrophy of the frontal, temporal, and parietal regions of the left hemisphere of the brain. PPA is a heterogeneous condition; the most prominent clinical feature is difficulty with language, while other cognitive domains are not affected at onset or in the early stages of the disease [5]. In 2011, an international group of experts proposed recommendations for PPA diagnosis and classification [6]. According to those recommendations, there are three main PPA variants: the nonfluent/agrammatic variant (nfvPPA), the semantic variant (svPPA), and the logopenic variant (lvPPA).

At least one of the following core features must be present to detect nfvPPA: (1) effortful, halting speech with inconsistent speech sound errors and distortions (apraxia of speech) and/or (2) agrammatism in language production. Moreover, at least two of the following features must also be present: (1) impaired comprehension of syntactically complex sentences, (2) spared single-word comprehension, and/or (3) spared object knowledge [6]. Imaging abnormalities in the left posterior frontoinsular region support the diagnosis of nfvPPA. Meanwhile, svPPA is a clinical syndrome caused by atrophy of the temporal lobes, leading to the selective impairment of semantic memory. The following core features must be present to establish a diagnosis of svPPA: (1) impaired confrontation naming and (2) impaired single-word comprehension. Moreover, at least three of the following features must also be present: (1) impaired object knowledge, (2) surface dyslexia or dysgraphia, (3) spared repetition, and/or (4) spared speech production (grammar and motor speech) [6]. Finally, lvPPA, the most recently identified PPA variant, is caused by predominant left posterior perisylvian or parietal atrophy. According to clinical criteria established in 2011, following core features are essential to the diagnosis of lvPPA: (1) the presence of anomia in spontaneous speech and (2) confrontation naming and impaired repetition of sentences and phrases [6]. At least three of the following features must also be present: (1) production of phonological errors, (2) preservation of semantic memory, (3) preservation of articulation and prosody, and/or (4) absence of frank agrammatism.

The initial evaluation is the first significant step toward the clinical management of dementia, and it is based on consensual diagnostic criteria. In some dementia syndromes, such as PPA, language deficit characterization is of major importance for the differential diagnosis. A language function assessment is part of the general diagnosis process for neurodegenerative diseases affecting language, and it generally includes medical history, mental status tests, physical and neurological exams, diagnostic tests, and brain imaging. The present article focuses on the contribution of a specific assessment of language abilities to the differential diagnosis of PPA. We first briefly present the cognitive neuropsychological approach to language assessment. Then, in sections addressing the main domains of language (i.e., spoken production, comprehension, written language), we briefly present the cognitive processes involved in the assessment tasks, the typical characteristics of each PPA variant, and common pitfalls in the interpretation of the results.

## 2. The Cognitive Neuropsychological Approach to Language Assessment

Compared to the clinicopathological approach to assessment, which aims to identify the diagnostic label that best corresponds to the observed language deficits (e.g., anomia; agrammatism), the cognitive neuropsychological approach aims to identify the impaired and preserved language abilities and localize their functional underlying origin [7]. This approach is derived from information processing theories in which cognitive functions, including language, are sustained by specialized, interconnected processing components. The assessment is conceived as an investigation based on the administration of specific tests in which stimuli are controlled or manipulated for psycholinguistic variables (e.g., length, frequency, familiarity) that are known to influence language processes. Error analysis in these tests is another source of information. For example, anomia may arise from distinct underlying deficits (e.g., in the activation of conceptual semantic representations or the retrieval of phonological forms of words in the lexicon), leading to distinct types of errors (e.g., semantic substitutions, phonemic errors). The main advantage of this assessment approach is that it goes beyond the mere description and classification of clinical syndromes and identifies impaired vs. preserved cognitive and linguistic components and processes. Furthermore, with a comprehensive portrait of the patient’s communication abilities, the clinician can better tailor the behavioral treatment to address impaired language processes with a restorative or compensatory objective.

## 3. The Assessment of Spoken Production

According to cognitive models of spoken word production [8], words are retrieved and produced through the activation of specialized and interconnected components. In these models, word production is conceived as a staged process in which the activation flow is initiated in a conceptual-semantic component, continues through the activation of phonological lexical representations, and ends with the execution of articulation mechanisms. Spoken production processes are usually assessed with tests exploring the ability to retrieve words in long-term memory (e.g., picture naming); repeat words, nonwords, and sentences [9]; and provide information in a discourse, conversational exchange, or interview [10]. A summary of the underlying cognitive deficits of language impairment and the salient characteristics of spoken production disorders in the three PPA variants are presented in Table 1.

### 3.1. The Assessment of Word Production

The easiest way to assess the ability to retrieve and produce spoken words is through picture naming tests, such as the Boston Naming Test in English [11] or the TDQ30 in French [12]. Theoretical models of spoken word production can facilitate the identification of the functional origins of deficits. These deficits may result from a loss of semantic representations or difficulty retrieving them. A breakdown at this level leads to semantic-based anomia. This is the case in svPPA; difficulty retrieving words manifests as no responses, semantic paraphasias, or vague circumlocutions [13]. In this variant, performance in naming tasks may be influenced by the familiarity and semantic category of the concepts (e.g., better or worse performance for natural vs. man-made concepts) as well as the visual complexity of the pictures due to possible concomitant associative visual agnosia [14].

Disruption in the activation of the phonological forms of words is responsible for the production of phonological errors in lvPPA [15] and nfvPPA [16]. Furthermore, in these variants, anomia manifests as no responses and specific circumlocutions. Studies have also shown that phonological short-term memory impairment contributes to spoken word production impairment in lvPPA, e.g., [15]. This process is responsible for the temporary storage of activated phonological representations until the actual execution of articulation mechanisms. In this case, performance in picture naming might be influenced by word length. Finally, impairment of the phonetic encoding stage, in which a motor representation for articulation is generated, leads to apraxia of speech in nfvPPA [17,18]. The confounding variables at this processing stage are syllable complexity [19].

In picture naming, the error type makes it easy to distinguish svPPA from the other variants. However, for an accurate differential diagnosis, differentiation between the phonological errors (i.e., substitution, deletion, displacement, and addition of phonemes) produced in both nfvPPA and lvPPA, and the phonetic errors (i.e., production of distorted phonemes and alteration of transitions between phonemes) associated exclusively to apraxia of speech in nfvPPA, is essential. To this respect, the advantage of the cognitive approach lies in the ability to differentiate the underlying origin of phonological errors, being the phonological short-term memory in the vlAPP and the activation of motor representations for articulation in the nfvAPP.

### 3.2. The Assessment of Repetition Abilities

The assessment of repetition abilities plays an important role in PPA differential diagnosis. For single words, these seemingly simple abilities involve linguistic processes devoted to the auditory analysis of stimuli and the activation of their lexical and semantic representations, followed by the activation of spoken production processes, including the maintenance of phonological forms of words in short-term memory. Episodic memory and semantic memory are added to these processes for the repetition of sentences [20]. For nonsense stimuli, such as nonwords or pseudowords, theoretical models of spoken production include a nonlexical/semantic route, which links auditory analysis to phonological short-term memory through an auditory-to-phonological conversion route.

Word and nonword repetition is usually preserved in svPPA, while the performance of individuals with nfvPPA is affected by apraxia of speech and marked by the production of phonological and phonetic errors [21]. The performance of these tasks by individuals with lvPPA is also affected; specifically, the production of phonological errors increases as word and/or nonword length increases [22]. Finally, sentence repetition is particularly important for PPA assessment, especially with respect to the core criteria proposed by Gorno-Tempini et al. for lvPPA diagnosis [6]. Sentence repetition is impaired in the three PPA variants, although there are distinct manifestations and severity levels. Individuals with lvPPA show significant impairment in sentence repetition, with performance negatively influenced by stimulus length but not by syntactic complexity [23]. In this task, their performance is notably marked by word omissions, semantic substitutions (replacement of one or more sentence words with words having similar or closely similar meanings), and phonological errors [24]. This profile is attributed to phonological short-term memory impairment. Meanwhile, sentence repetition is mildly impaired in svPPA [21]. In this variant, performance is not dependent on sentence length or syntactic complexity but on comprehension of the words of the sentence [25]. Finally, in nfvPPA, sentence repetition is disrupted due to impairment of the phonetic encoding stage of spoken production [26]. A deficit in the rehearsal mechanism of encoded verbal information has also been suggested to explain the production of phonological errors in sentence repetition in nfvPPA [21]. In this variant, performance might be influenced by the syntactic complexity of sentences [27] due to associated agrammatism [28].

To conclude, although repetition is disrupted in the three PPA variants (except for single words and nonwords in svPPA), the functional origins of the impairments provide essential clues for the differential diagnosis. However, a prerequisite for this is the use of adequate tests in which confounding variables, such as stimulus length and syntactic complexity, are controlled and manipulated.

### 3.3. The Assessment of Language Production in Spontaneous Speech and Narrative Discourse

Traditional tests provide useful information on linguistic abilities and language impairments in PPA. However, performance on these tests does not necessarily predict how a person will communicate in more naturalistic settings and everyday life. Functionally, spontaneous speech is the best way to appreciate the verbal and nonverbal communication of individuals with PPA. This simple everyday life ability involves the execution and interaction of various cognitive (episodic memory, semantic memory, short-term memory, working memory, executive functions, attentional ability) and linguistic (speech production, speech comprehension, pragmatics) processes [29], making it particularly vulnerable in PPA. The functional origin of language deficits in the three PPA variants manifests in different ways in spontaneous speech. Spontaneous speech in patients with svPPA is fluent, well-articulated, and grammatically correct; semantic impairment primarily causes word-finding difficulties in the form of aborted sentences, latencies, circumlocutions, and occasional semantic paraphasias [30]. In nfvPPA, phonetic encoding impairment makes spontaneous speech slow, hesitant, and effortful [31]. As the disease progresses, speech fluency decreases, and articulation and prosody become more affected. Moreover, disruption in the activation of the phonological forms of words is responsible for hesitations and the production of phonological errors and contributes to the slow rate and abnormal pauses in connected speech [31]. Agrammatism, the second core feature of nfvPPA, can be subtle and may go unnoticed in connected speech. When apparent, agrammatism in spontaneous speech is marked by difficulty with inflecting verbs, the omission or substitution of closed-class words, and difficulty in sentence construction [32]. Finally, in lvPPA, impairment is localized in the activation of phonological forms and phonological short-term memory and causes anomia and the production of phonological errors in spontaneous speech.

However, it is important to be aware that spontaneous speech may not be particularly useful for examining linguistic variables such as word retrieval and morphosyntax because deficits can be masked when individuals manipulate the complexity of their utterances and the specific lexical items they select. By contrast, narrative tasks such as storytelling or scene description which constrain an individual to certain vocabulary items and discourse structures can be highly informative over and above unconstrained conversation. In these tasks, similar manifestations to those mentioned previously for spontaneous speech might be observed in the three variants of PPA.

## 4. The Assessment of Comprehension

In PPA, the assessment of comprehension usually includes tests that explore word and sentence comprehension as well as object knowledge, which is stored in semantic memory. For differential diagnosis, the assessment of oral comprehension is usually sufficient. However, in the presence of noncompensated hearing loss, a written assessment can be useful. A summary of the salient comprehension deficits and their underlying cognitive impairment in the three PPA variants are presented in Table 2.

### 4.1. The Assessment of Word Comprehension and Object Knowledge

The assessment of word comprehension is crucial to the differential diagnosis of PPA. Word–picture matching tests, such as the Peabody Picture Vocabulary Test [33] in English or the spoken word-to-picture matching subtest of the BECLA battery [34] in French, are usually used to assess single-word comprehension. From the cognitive neuropsychology point of view, the processes involved in these tests include auditory analysis of the stimulus, activation of its lexical representation, and activation of the corresponding semantic representation within the semantic memory. Single-word comprehension is usually well-preserved in lvPPA and nfvPPA, whereas its impairment is one of the core features in svPPA [6]. In this variant, the deficit arises directly from semantic memory impairment, and errors are mostly made on semantic distractors.

The assessment of object knowledge directly recruits the activation of semantic information in semantic memory as well as links between semantic concepts. Picture association tasks, such as the Pyramids and Palm Trees Test [35], are usually used to assess nonverbal semantic processing. However, when visual impairment or visual agnosia is present, tests that use written words, such as the written word-to-written word semantic matching subtest of the BECLA battery, are preferable [34]. In the presence of a semantic memory deficit, semantic access to both pictures and words should be impaired. It is important to consider that this type of task can be especially challenging in the presence of executive deficits, which are often found in PPA, and could lead to misleading results. In this case, a simpler task, such as a semantic questionnaire (e.g., QueSQ in French [36]) can be used. Given the core impairment of semantic memory, object knowledge in nonverbal and verbal modalities is impaired in svPPA. However, it is usually largely spared in lvPPA and nfvPPA.

Psycholinguistic factors, such as familiarity and typicality, are particularly important when it comes to semantic memory. In svPPA, word comprehension and object knowledge performance are usually better preserved for concepts that are familiar to the person (e.g., objects used daily) [37]. Typicality is also important, as more typical items of a semantic category (e.g., apple) are processed faster than less typical items of the same category (e.g., mango) [38].

### 4.2. The Assessment of Sentence Comprehension

Sentence comprehension is usually assessed using a sentence-picture matching task, such as the Northwestern Assessment of Verbs and Sentences in English [39] or the Batterie d’évaluation de la compréhension syntaxique [40] in French. Sentence comprehension is typically well preserved in svPPA (if all words in the sentence are understood) and lvPPA [41]. However, in lvPPA, phonological short-term memory impairment could lead to difficulty understanding long sentences [41]. In this context, assessing sentence comprehension using written material could be particularly relevant for differential diagnosis, as visual support is likely to reduce the load on phonological short-term memory and lead to better performance. In nfvPPA, sentence comprehension is impaired, particularly for syntactically complex sentences [42]. In addition to the core impairment of grammar, working memory deficits have been documented in this clinical population [41] and could contribute to difficulties with syntactically complex sentences. Syntax complexity (e.g., active, passive, relative sentences) and sentence length are key parameters and should be controlled or manipulated in sentence comprehension tests.

## 5. The Assessment of Written Language

The assessment of written language involves the administration of reading and written spelling tests using different types of material: words, nonwords, sentences, texts, and narrative discourse. A summary of the underlying cognitive deficits of written language impairment and the salient characteristics of reading and written spelling disorders in the three PPA variants are presented in Table 3.

### 5.1. The Assessment of Reading Abilities

In cognitive models, such as the dual-route cascaded model [43], reading is mediated by the computation of orthographic, phonological, and semantic information via two distinct routes: the lexical-semantic route and the sublexical route. The lexical-semantic route involves reading words with inconsistent orthography-to-phonology mappings (e.g., *yacht*), while the sublexical route, mediated by grapheme-to-phoneme conversion rules, mainly involves reading words with consistent orthography-to-phonology mappings (e.g., *banana*). Therefore, the control and manipulation of psycholinguistic variables of stimuli are essential in reading tests. According to the dual-route cascaded model, impairment of the lexical-semantic route alone causes surface dyslexia, which is characterized by difficulty reading inconsistent words [44]. Meanwhile, impairment of the sublexical route alone results in phonological dyslexia [45]. However, the disruption of both reading pathways results in deep dyslexia, which is characterized by difficulty reading words and nonwords and the production of semantic and visual paralexias [46]. Differentiation between the two reading pathways is particularly important for the differential diagnosis of PPA.

Semantic memory impairment affects the lexical-semantic route of reading in individuals with svPPA. This impairment causes surface dyslexia, one of the clinical features of this variant [6]. These individuals are better at reading orthographically consistent words than inconsistent words, and they show a preserved ability to read nonwords. Most of their reading errors consist of regularizations (e.g., bread→/brid/). Their performance is also influenced by the lexical frequency of words [47] and is directly linked to the extent of semantic loss [48]. Reading ability impairment is not part of the clinical criteria for nfvPPA or lvPPA. Although reading abilities are considered to be preserved in nfvPPA, impairment may emerge with disease progression [49]. Reading is characterized by phonological dyslexia, which is specifically affected by unfamiliar words and nonwords and marked by the production of phonological errors [50]. This profile suggests impairment of the sublexical route of reading. Reading is also characterized by manifestations of apraxia of speech in nfvPPA. In lvPPA, the underlying impairment of the activation of phonological lexical representation and phonological short-term memory causes difficulty in reading words and nonwords. Errors consist of a mix of phonological, semantic, and visual paralexias, which is suggestive of impairment of both routes of reading (deep/phonological dyslexia) [51]. The overlap of the manifestations makes it difficult to make a differential diagnostic between nfvPPA and lvPPA based on reading impairment alone. The manifestations of reading impairment in the three PPA variants are qualitatively similar but can be quantitatively exacerbated when abilities are tested with sentences or texts.

### 5.2. The Assessment of Writing Abilities

Cognitive models of writing, such as the dual-route model, also involve two distinct routes: the lexical-semantic route and the sublexical route [52]. The lexical-semantic route is used to write familiar words and includes processes that are initiated in the conceptual-semantic component, continue with the activation of orthographic lexical representations, and end with the execution of writing mechanisms. In a writing-to-dictation task, this sequential process is preceded by recognition of the spoken word in the phonological lexicon. The sublexical route is used to write unfamiliar words and nonwords through the activation of phonological-to-orthographic conversion rules. Impairment of the lexical-semantic route alone causes surface agraphia in which the use of the sublexical route leads to the production of phonologically plausible errors (e.g., phone → FONE). When only the sublexical route is disrupted, the resulting deficit, which is called phonological agraphia, affects nonword spelling. Finally, impairment of both routes causes deep agraphia, which is characterized by difficulty writing words and nonwords and the production of semantic and visual errors. Phonologically implausible errors (i.e., insertions, deletions, or transpositions of letters) are also possible due to partially impaired access to orthographic word forms.

Surface agraphia is one of the clinical features of svPPA [6]. The deficit is caused by impairment of the lexical-semantic route and is directly linked to semantic loss and difficulty activating orthographic forms in the lexicon [53]. The impairment is apparent regardless of the nature of the written task (e.g., spontaneous writing, writing-to-dictation, picture naming). Written production is usually more impaired than reading in nfvPPA. In this variant, writing impairment is suggestive of deep agraphia [54] due to difficulty retrieving orthographic forms of words in the lexicon, combined with disruption of the sublexical route [55]. Writing performance is also negatively influenced by orthographic inconsistency in nfvPPA but to a lesser degree than in svPPA [56]. The production of phonologically plausible errors is not exceptional in this variant [57]. Agrammatism is also generally apparent in nfvPPA in narrative discourse and spontaneous writing [58]. Finally, patients with lvPPA usually present with phonological agraphia, which is characterized by an impaired sublexical route and partially impaired access to orthographic word forms in the lexical-semantic route [55]. Other forms of agraphia, such as surface agraphia, are also possible in lvPPA [54]. As with reading, there is a partial overlap in the manifestations of agraphia in nfvPPA and lvPPA.

## 6. Conclusions

As shown in this article, PPA is a heterogeneous syndrome in terms of its clinical manifestations. The aforementioned diagnostic criteria aid in the differentiation of the three PPA variants [6]; however, they are very broad and are the subject of controversy. Their limits are important and relate to various aspects of linguistic semiology [59]. For example, Sajjadi et al. [60] performed a factor analysis of the language tasks results of 46 patients with PPA. The results were consistent with the existence of two variants, one characterized by semantic deficits (23% of cases) and the other by agrammatism and apraxia of speech (26% of cases). However, the analysis did not identify a cluster of measures that were compatible with the clinical profile of lvPPA. A few years later, Hoffman et al. [61] reanalyzed the data from those patients without taking their initial clinical diagnoses into account; they identified a distinct cluster for svPPA but not for the other variants.

We have also shown that an assessment process based on cognitive neuropsychological models allows clinicians to understand patients’ deficits (i.e., surface manifestations, underlying origins, affected components) and identify strengths and weaknesses in their communication abilities. Although some surface manifestations of language impairments might overlap (e.g., anomia, repetition deficits) in PPA, their underlying origins are different and differentiable. Therefore, the cognitive approach to language assessment is useful for the differential diagnosis of PPA. Future studies should extend beyond the surface manifestations of language impairment in PPA to develop more comprehensive and distinctive diagnostic criteria.

In addition to its importance for the differential diagnosis of PPA, identifying the underlying cognitive deficit of the language impairment is crucial in order to plan effective therapeutic interventions based on restorative and compensatory approaches or teach communication strategies to patients and their relatives. For example, anomia is often targeted in PPA behavioral treatments. However, the underlying cause of anomia is functionally localized in the activation of phonological forms in the lexicon in lvPPA and nfvPPA, while it is caused by an impairment of semantic memory in svPPA [6]. The exact origin of anomia has an important role to play in how the intervention is planned. For example, when semantic memory is impaired, generalization is limited, as relearning primarily relies on episodic memory [62]. Moreover, treatment success in svPPA has been shown to be related to residual semantic knowledge and contextual information, which were more preserved for significant and familiar words [63]. Therefore, the selection of vocabulary based on personal interests is more crucially important in svPPA than in the two other PPA variants [64].

Identifying the underlying deficit is also important in compensatory approaches. For example, the choice of an app to compensate for language impairments depends directly on their functional origin. An app in which the content is organized by semantic categories (e.g., fruits/vegetables/meat) could be very effective in compensating for lexical-based anomia in lvPPA and nfvPPA. In contrast, teaching a patient with svPPA to search for information on the Internet to cope with comprehension problems (e.g., Wikipedia, Google Images) using keywords would be preferable due to the semantic origin of his/her difficulties [65].

Finally, the underlying deficit must also be considered when teaching communication strategies to patients and their relatives. For example, one popular strategy to compensate for word-finding difficulty is to encourage the patient to describe the object that he/she is unable to produce (e.g., Mug: What I use to drink coffee). However, while this could be a very efficient strategy for an impairment in the activation of phonological forms, it would be ineffective for an impairment that is localized in semantic memory because the spontaneous generation of a useful or reliable definition of the word would be compromised due to difficulty activating conceptual knowledge.

The aforementioned examples mainly concern word retrieval deficits. It is worth noting that the cognitive approach is similarly useful for treating, compensating, or teaching communication strategies for other deficits associated with PPA, such as agrammatism [66], apraxia of speech [67] and spelling deficits [68]. In summary, considering the underlying deficit in the clinical management of PPA allows for a tailored intervention that is likely to maximize benefits for patients and their relatives.

## Figures and Tables

**Table 1:** Underlying cognitive deficits and salient characteristics of spoken production disorders in the three PPA variants.

Spoken Production	svPPA	nfvPPA	lvPPA
Underlying deficit	Semantic memory	- Lexicon: Activation of phonological forms - Phonetic encoding: Activation of motor representations for articulation	- Lexicon: Activation of phonological forms - Phonological short-term memory
Influence of psycholinguistic variables	- -Concept familiarity - -Semantic category * - -Visual complexity of pictures *	- -Syllable complexity - -Stimulus length - -Syntactic complexity	- Stimulus length
Word production: Picture naming	Impaired: No responses, semantic paraphasias, and vague circumlocutions	Impaired: Apraxia of speech, phonological errors, no responses, and specific circumlocutions	Impaired: Phonological errors, no responses, and specific circumlocutions
- Repetition - Words - Nonwords - Sentences	- -Preserved - -Preserved - -Mild impairment	Impaired for all types of stimuli: Apraxia of speech, phonological errors	Impaired for all types of stimuli: Phonological errors
Spontaneous speech and narrative discourse	Word-finding difficulties: Aborted sentences, latencies, circumlocutions, and occasional semantic paraphasias	Slow, hesitant, and effortful; phonetic and phonological errors; and agrammatism	Impaired: Word-finding difficulties and phonological errors

* Potential but nonessential psycholinguistic variable.

**Table 2:** Underlying cognitive deficits and salient characteristics of comprehension disorders in the three PPA variants.

Comprehension	svPPA	nfvPPA	lvPPA
Underlying deficit	Semantic memory	Grammar and working memory	Phonological short-term memory
Influence of psycholinguistic variables	- Concept familiarity - Concept typicality - Semantic category * - Visual complexity of pictures *	- Syntactic complexity	- Sentence length
Word comprehension	Impaired: Errors on semantic distractors	Preserved	Preserved
Object knowledge	Impaired in verbal and nonverbal modalities	Preserved	Preserved
Sentence comprehension	Preserved	Impaired in syntactically complex sentences (e.g., passive and relative sentences)	Preserved but can be impaired in long sentences

* Potential but nonessential psycholinguistic variable.

**Table 3:** Underlying cognitive deficits and salient characteristics of written language disorders in the three PPA variants.

Reading	svPPA	nfvPPA	lvPPA
Underlying deficit	Lexical-semantic route: Semantic memory	- Lexical-semantic route: Activation of phonological forms - Sublexical route: Activation of grapheme-to-phoneme conversion rules	- Lexical-semantic route: Partial impairment in the activation of phonological forms - Sublexical route: Activation of grapheme-to-phoneme conversion rules
Influence of psycholinguistic variables	- Orthographic consistency	- Orthographic consistency - Lexicality: Words and nonwords - Lexical frequency	- Lexicality: Words and nonwords - Lexical frequency
Reading	Surface dyslexia: Regularization errors	Phonological dyslexia: Phonological errors and impact of apraxia of speech	Mixed (deep/phonological) dyslexia: Phonological, semantic, and visual paralexias
**Writing**			
Underlying deficit	Lexical-semantic route: Semantic memory	- Lexical-semantic route: Activation of orthographic forms - Sublexical route: Activation of phonological-to-orthographic conversion rules	- Lexical-semantic route: Partial impairment in the activation of orthographic forms - Sublexical route: Activation of phonological-to-orthographic conversion rules
Influence of psycholinguistic variables	- Orthographic consistency	- Orthographic consistency - Lexicality: Words and nonwords - Lexical frequency	- Lexicality: words and nonwords - Lexical frequency
Word production: Picture naming and writing-to-dictation	Surface agraphia: No responses, semantic paragraphias, and phonologically plausible errors	Mixed agraphia: Phonologically and nonphonologically plausible errors	Phonological agraphia: Nonphonologically plausible errors and possible phonologically plausible errors
Spontaneous writing	Word-finding difficulties and surface agraphia: Aborted sentences, phonologically plausible errors, and occasional semantic paragraphias	Mixed agraphia and agrammatism: Phonologically and nonphonologically plausible errors and syntactic errors	Phonological agraphia: Nonphonologically plausible errors and possible phonologically plausible errors
